# Angiotensin converting enzyme gene polymorphism in type 2 diabetics with nephropathy

**DOI:** 10.4103/0971-4065.65314

**Published:** 2010-04

**Authors:** V. V. S. Naresh, A. L. K. Reddy, G. Sivaramakrishna, P. V. G. K. Sarma, R. V. Vardhan, V. S. Kumar

**Affiliations:** Department of Nephrology, Sri Venkateswara Institute of Medical Sciences, Sri Venkateswara University, Tirupati - 517 507, India; 1 Department of Biotechnology, Sri Venkateswara Institute of Medical Sciences, Sri Venkateswara University, Tirupati - 517 507, India; 2 Department of Statistics, Sri Venkateswara Institute of Medical Sciences, Sri Venkateswara University, Tirupati - 517 507, India

Sir,

We thank the reader for his observations and the editor for having given us the opportunity to offer our explanation.

1. The answer to the query that the genetic polymorphism studies should be performed on a population scale is – We agree with the observation; although desirable owing to the financial constraints involved, we could not undertake the study at a population level. However, the statistical relevance of the data gathered and its possible extrapolation to a larger group has been ensured by using appropriate statistical methods while interpreting the results.
2. The answer to the query that race might have an implication on the results is – We again agree with the observation, the same having been reflected in earlier studies from different geographical locations including those from the Indian subcontinent, although with conflicting results. Our study was performed with the intention to gather data from patients of South Indian ethnicity in whom the prevalence of type 2 diabetes mellitus is high.[1–3]
3. The answer to the query that correlation between ACE polymorphism and diabetic retinopathy showed null correlation in different studies is – We restricted our study only to investigating the relationship of ACE gene polymorphism and diabetic nephropathy in type 2 diabetes mellitus patients. Hence, are unable to comment on the mentioned observation of the reader with regard to our study group.
